# Kinematic and EMG data during underwater dolphin kick change while synchronizing with or without synchronization of kick frequency with the beat of a metronome

**DOI:** 10.1016/j.dib.2017.07.027

**Published:** 2017-07-15

**Authors:** Keisuke Kobayashi Yamakawa, Hirofumi Shimojo, Hideki Takagi, Shozo Tsubakimoto, Yasuo Sengoku

**Affiliations:** a Department of Sport Wellness Sciences, Japan Women's College of Physical Education, 8-19-1, Kitakarasuyama, setagaya-ku, Tokyo, Japan; b Department of Health and Sports, Niigata University of Health and Welfare, 1398 shimami-cho, Kita-ku, Niigata city, Niigata, Japan; c Faculty of Health and Sport Sciences, University of Tsukuba, 1-1-1, Tennoudai, Tsukuba, Ibaraki, Japan

## Abstract

We investigated the effects of synchronizing kick frequency with the beat of a metronome on kinematic and electromyographic (EMG) parameters during the underwater dolphin kick as a pilot study related to the research that entitled “*Effect of increased kick frequency on propelling efficiency and muscular co-activation during underwater dolphin kick”* (Yamakawa et al., 2017) [1]. Seven collegiate female swimmers participated in this experiment. The participants conducted two underwater dolphin kick trials: swimming freely at maximum effort, and swimming while synchronizing the kick frequency of maximum effort with the beat of a metronome. The kinematic parameters during the underwater dolphin kick were calculated by 2-D motion analysis, and surface electromyographic measurements were taken from six muscles (rectus abdominis, erector spinae, rectus femoris, biceps femoris, tibialis anterior, and gastrocnemius). The results revealed no significant differences in the kinematic and EMG parameters between trials of the two swimming techniques. Therefore, the action of synchronizing the kick frequency with the beat of a metronome did not affect movement or muscle activity during the underwater dolphin kick in this experiment.

## **Specifications Table**

**Table:** 

Subject area	*Biomechanics*
More specific subject area	*Motion analysis*
Type of data	*Tables*
How data was acquired	*Kinematic analysis using 2-D direct Linear Transformation; Surface EMG*
Data format	*Analyzed*
Experimental factors	*Conditions: two underwater dolphin kick trials which are swimming freely at maximum effort and swimming while synchronizing the kick frequency of their maximum effort with the beat of a metronome.*
Experimental features	*Video of the swimming action and the surface EMG of the six muscles (rectus abdominis, erector spinae, rectus femoris, biceps femoris, tibialis anterior, and gastrocnemius) were simultaneously recorded during each trial.*
Data source location	*Tsukuba, Ibaraki, Japan*
Data accessibility	*All data are available with this article.*
Related research article	*Yamakawa KK, Shimojo H, Takagi H, Tsubakimoto S, and Sengoku Y. Effect of increased kick frequency on propelling efficiency and muscular co-activation during underwater dolphin kick. Human movement Science. 2017 54: 276–286.*

## **Value of the data**

• Data comparing kick frequency with or without synchronization to the beat of a metronome on kinematic and EMG parameters during the underwater dolphin kick are provided.
• There were no significant differences in the kinematic or EMG parameters between two trials which consisted of swimming freely at maximum effort and swimming while synchronizing the kick frequency of maximum effort with the beat of a metronome.
• Synchronizing kick frequency with the beat of a metronome does not affect movement or muscle activity during the underwater dolphin kick.

## 1 Data

We provide a dataset showing differences in the kinematic and EMG parameters between two underwater dolphin kick trials: swimming freely at maximum effort (Maximum effort) and swimming while synchronizing the kick frequency of maximum effort with the beat of a metronome (Synchronized 100% frequency). No significant differences between the two trials were detected in any kinematic or EMG parameters (Tables 1 and 2 below).

**Table 1:** Kinematic parameters during the two underwater dolphin kick trials: swimming freely at maximum effort (Maximum effort) and swimming while synchronizing the kick frequency of maximum effort with the beat of a metronome (Synchronized 100% frequency).

Variables	Unit	Maximum effort	Synchronized 100% frequecy	*t*-test
				*p*-value
Average swimming velocity	m s^−1^	1.36±0.09	1.38±009	0.16
Kick frequency	Hz	2.25±0.13	2.31±0.28	0.50
Kick amplitude	m	0.42±0.03	0.41±0.05	0.71
Downward kick phase	%	48.0±1.6	48.8±1.5	0.27
Upward kick phase	%	56.4±1.7	55.7±1.4	0.28
Froude efficiency		0.71±0.02	0.71±0.02	0.95
Toe vertical velocity				
Downward kick phase				
– Average velocity	m s^−1^	-1.83±0.11	−1.83±0.11	0.79
– Maximum velocity	m s^−1^	-3.12±0.14	−3.12±0.14	0.99
Upward kick phase				
– Average velocity	m s^−^^1^	1.66±0.13	1.63±0.14	0.19
– Maximum velocity	m s^−1^	2.73±0.25	2.68±0.30	0.42

**Table 2:** EMG parameters during the two underwater dolphin kick trials: swimming freely at maximum effort (Maximum effort) and swimming while synchronizing the kick frequency of maximum effort with the beat of a metronome (Synchronized 100% frequency). Rectus abdominis, RA; Erector spinae, ES; Rectus femoris, RF; Biceps femoris, BF; Tibialis anterior, TA; Gastrocnemius, GAS.

Variables	Unit	Maximum effort	Synchronized 100% frequecy	*t*-test
				*p*-value
Average RMS				
RA	mV	0.137±0.170	0.142±0.032	0.64
ES	mV	0.100±0.025	0.100±0.030	0.99
RF	mV	0.143±0.043	0.140±0.040	0.75
BF	mV	0.162±0.051	0.159±0.048	0.66
TA	mV	0.084±0.027	0.093±0.027	0.08
GAS	mV	0.198±0.132	0.204±0.134	0.50
Active phase				
RA	%	66.2±5.8	68.0±6.3	0.56
ES	%	65.5±5.1	66.7±7.3	0.66
RF	%	66.0±6.3	64.0±8.5	0.50
BF	%	68.7±8.4	67.3±6.7	0.55
TA	%	85.9±4.9	85.4±6.9	0.89
GAS	%	67.1±10.7	65.8±4.3	0.79
Co-active phase				
RA-ES	%	31.8±9.8	35.4±10.6	0.47
RF-BF	%	35.0±14.2	31.6±13.8	0.51
TA-GAS	%	58.1±14.9	51.6±9.6	0.35

## 2 Experimental design, materials and methods

Seven collegiate female swimmers (mean±standard deviation (SD): age, 19.0±0.6 years; height, 1.64±0.05 m; weight, 56.7±3.3 kg) participated in this experiment. The experimental trials consisted of 15 m underwater dolphin kick swimming. Firstly, the participants swam freely using the underwater dolphin kick at maximum effort. Secondly, they swam using the underwater dolphin kick while synchronizing the kick frequency of their maximum effort with the beat of a metronome. A rest interval of at least five minutes was set between the two trials. For 2-D motion analysis, video of the sagittal movement was recorded by two cameras at a 100 Hz sampling rate. To evaluate muscle activity, the surface EMG of six muscles (rectus abdominis, erector spinae, rectus femoris, biceps femoris, tibialis anterior, and gastrocnemius) were measured using a wireless recorder with an 8-channel EMG logger (Biolog2, S&ME Inc., Japan). From all the collected data, the kinematic and EMG parameters were calculated according to the method described by Yamakawa et al. [1]. All data are reported as the mean and standard deviation (Mean±SD). Statistical analyses were conducted using BellCurve for Excel (SSRI Inc., Tokyo, Japan). The normality of all data was confirmed using the Shapiro-Wilk test. A paired *t*-test was used to compare the kinematic and EMG parameter data between the two conditions. The statistical significance level was set at 5% in this work (*P*<0.05).
